# The Mediating Role of Bioactive Molecules in Gut Microbiota–Bone Metabolism Crosstalk

**DOI:** 10.3390/nu17213421

**Published:** 2025-10-30

**Authors:** Xinping Liang, Luoyang Wang

**Affiliations:** Department of Immunology, School of Basic Medicine, Qingdao University, Qingdao 266071, China

**Keywords:** gut microbiome, osteoporosis, short-chain fatty acids, vitamin D, estrogen

## Abstract

The interaction between the gut microbiota and the skeletal system has evolved into a new research focus. Studies underscore the role of bioactive metabolites in sustaining systemic balance via the “gut microbiota–endocrine–skeleton” axis, where they modulate metabolic processes and organ morphology through intracellular signaling. A key bidirectional relationship exists with the gut: shifts in gut microbiota affect host metabolism and subsequent metabolite profiles, while these metabolites can, in turn, reshape the intestinal microenvironment. This review explores how short-chain fatty acids (SCFAs), estrogen, and vitamin D modulate osteoporosis via the gut–bone axis. It synthesizes evidence of their signaling pathways and metabolic roles, identifies research gaps from recent clinical studies, and evaluates gut microbiota-targeted therapeutic strategies for potential clinical translation.

## 1. Introduction

Emerging as an escalating global health challenge, osteoporosis compromises skeletal integrity in over 200 million individuals worldwide, imposing substantial socioeconomic burdens while significantly diminishing life quality [1]. This metabolic bone disorder manifests through progressive reductions in bone mineral density (BMD) and deterioration of trabecular architecture. Epidemiologically, this condition predominantly affects the elderly, with the highest prevalence in women over 55 and men over 65 [2]. Recent studies recognize diet and physical activity as key modifiable factors critically affecting bone mineral density in the elderly. This non-pharmacological strategy proves especially effective in countering postmenopausal osteoporosis resulting from reduced sex hormone levels [3]. Current evidence confirms that sex steroid hormones, notably estrogens and androgens, are central to osteoporosis pathogenesis, as demonstrated by well-established ovariectomized (OVX) and orchiectomized (ORX) murine models [4,5]. The clinical relevance is heightened in postmenopausal women, where estrogen-deficient osteoporosis accounts for most diagnosed cases [6]. Growing research now highlights gut microbiota changes linked to sex hormone deficiency, uncovering potential key mechanisms in osteoporosis progression.

As the most expansive and intricately organized microecosystem in human physiology, the gastrointestinal tract hosts an astonishing 10^14^ microorganisms [7], comprising bacteria, fungi, viruses, and archaea in a sophisticated symbiotic partnership with their host. This microbial consortium primarily consists of five major phyla: Firmicutes, Bacteroidetes, Actinobacteria, Proteobacteria, and Verrucomicrobia. The gut microbiota composition differs significantly between adults and infants, and both demonstrate an ability to regulate osteoporosis development [5,8,9]. This age-specific influence is further evidenced by the finding that transplanting microbiota from one age group to another preserves its distinct regulatory effects in the recipient [10]. Overall, the gut microbiome profoundly influences bone metabolism across all life stages, suggesting its potential for optimizing current osteoporosis treatments [11,12,13].

**Figure 1:**
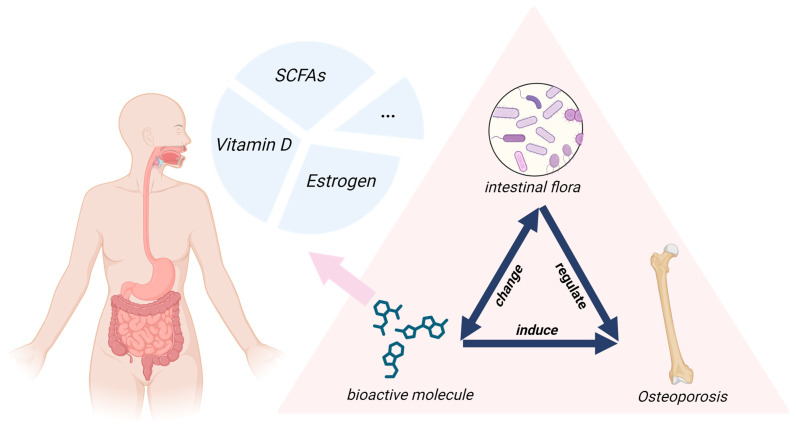
Schematic diagram illustrating the proposed interactions among osteoporosis, gut microbiota, and bioactive compounds. The arrows highlight the specific categories of bioactive compounds reviewed herein. Created in BioRender. Liang, Xinping (2025), https://BioRender.com/o5x7yho (accessed on 2 June 2025).

Recent studies show a complex two-way relationship between the gut and bones, called the “gut–bone axis.” Certain bioactive molecules can improve gut health, change the microbiome, affect bone mass, and alter gut permeability [14,15]. The microbiome is now a key target for bone health, and the gut–bone axis offers a promising way to prevent and treat osteoporosis [16,17]. However, most earlier reviews have largely focused on single metabolites or isolated mechanisms, lacking an integrated analysis of multi-signal networks—especially the synergistic interactions among SCFAs, estrogen, and vitamin D. To address this gap, this review is structured around three key mediators: SCFAs, estrogen, and vitamin D. It synthesizes recent evidence on their roles in the gut–bone axis, examining their associated signaling pathways, their impact on the gut microbiota, and relevant clinical evidence (Figure 1). This integrated approach clarifies their interactions within a triangular network connecting gut microbiota, endocrine signaling, and bone homeostasis. It examines these metabolites as receptor-mediated signaling molecules and explores their tripartite relationship in regulating the microbial ecosystem and maintaining skeletal integrity [18,19]. By synthesizing evidence from animal and clinical studies, this review elucidates key mechanisms in the gut-microbiota-bone pathway and proposes novel microbiome-targeted strategies. This work addresses critical evidence gaps and provides a foundation for advancing personalized osteoporosis management.

## 2. Short-Chain Fatty Acids (SCFAs)

The role of SCFAs in the pathogenesis and therapeutic mechanisms of osteoporosis-related disorders has gained increasing recognition. Significant attention has been directed toward quantitative alterations in SCFA-producing microbiota during osteoporotic progression. Akinsuyi and Roesch reanalyzed five publicly available 16S rRNA partial sequence datasets, revealing decreased abundance of SCFA producers in the osteoporosis (OP) group compared to healthy controls (HC) [20]. Furthermore, compelling evidence suggests that Tenericutes, a bacterial phylum exhibiting strong correlation indices with SCFA synthesis pathways, is associated with reduced fracture risk [11]. These collective findings cement SCFAs’ pivotal role in orchestrating microbiome-osseous tissue crosstalk. Among SCFAs, propionate and butyrate emerge as particularly significant components. Propionic acid (PA) exerts dual anti-inflammatory and osteoprotective effects, showing clinical relevance in multiple sclerosis (MS) patients with concomitant OP. PA supplementation beneficially modulates serum β-CrossLaps and osteocalcin levels, manifesting as conspicuous reductions in bone resorption marker β-CrossLaps post-treatment [21]. Butyrate prevents postmenopausal and inflammation-related bone loss. It can inhibit the secretion of inflammatory cytokines and alter the number of Treg cells. Crucially, marrow-resident Tregs activate CD8+ T cells to secrete the osteogenic Wnt ligand Wnt10b, thereby initiating the Wnt/β-catenin signaling cascade that drives bone formation [22,23]. Enhanced butyrate production has been shown to upregulate tight junction protein expression [24] and improve intestinal barrier integrity and function, consequently ameliorating periodontal and other bone loss conditions [25]. These findings suggest that investigations into butyrate’s anti-osteoporotic mechanisms should consider both immune modulation and gut barrier enhancement. In addition, studies employing R26STAT3Cstopfl/fl CD4Cre murine osteoporosis models reveal that SCFAs retain inhibitory effects on osteoclast differentiation even at minimal serum concentrations, while simultaneously exerting direct impacts on bone marrow progenitor cells [26]. This phenomenon appears linked to SCFA-mediated metabolic reprogramming of osteoclasts through TRAF6 and NFATc1 downregulation, effectively suppressing osteoclastogenesis and bone resorption [27]. Collectively, these findings highlight the remarkable regulatory potential of SCFAs in both osteoblast and osteoclast formation/differentiation processes.

### 2.1. Endocrine Signaling Pathways Mediating SCFAs-Induced Osteoporosis Alleviation

As endogenous signaling molecules with bioactivity, SCFAs exert their anti-osteoporotic effects primarily through targeted receptor-ligand crosstalk. Within the intestinal milieu, SCFAs exhibit potent regulatory capacity by suppressing the synthesis of Receptor Activator of Nuclear Factor Kappa-B Ligand (RANKL) [28]. This inhibition proves critical, as RANKL-mediated osteoclastogenesis depends on its interaction with the RANK receptor to activate NF-κB signaling cascades. Studies further show that RANKL–RANK interaction triggers ubiquitination and degradation of the Pregnane X Receptor (PXR), diminishing the binding of residual PXR to P65 and thereby promoting osteoclast overactivation in estrogen-deficient conditions [20,29]. Moreover, moderate activation of NF-κB helps maintain intestinal epithelial barrier integrity [30]. The RANKL-RANK engagement alters gut conditions by stimulating mitogenic signaling in intestinal epithelial cells, leading to villus hypertrophy and luminal surface expansion [31]. Such gastrointestinal remodeling may synergistically optimize systemic mineral homeostasis, ultimately exerting indirect beneficial effects on skeletal metabolism.

**Figure 2:**
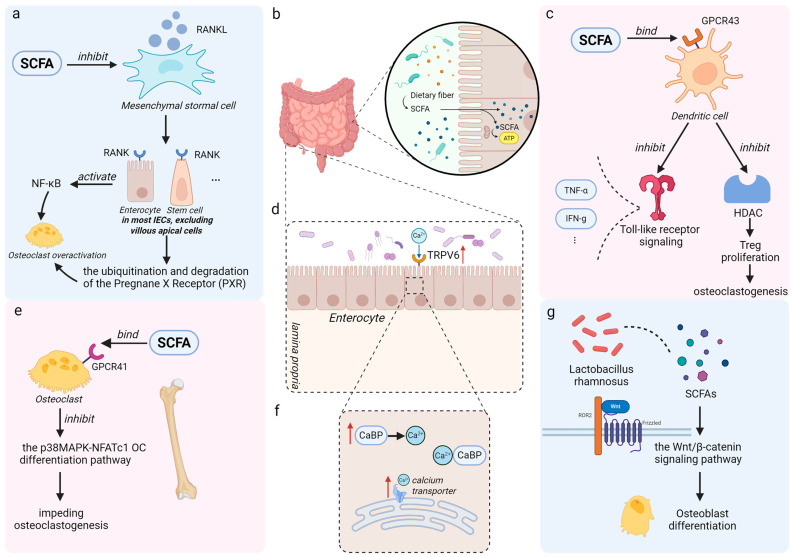
(**a**–**g**) Mechanism of short-chain fatty acids relieving osteoporosis. Created in BioRender. Liang, Xinping (2025), https://BioRender.com/rzrmiir (accessed on 2 June 2025).

At the same time, SCFAs engage in sophisticated molecular dialogues with G protein-coupled receptors (GPCRs), the predominant family of transmembrane signaling hubs, including key subtypes such as GPR41, GPR43, and GPR109A. This interaction activates signaling cascades that regulate immune functions, promotes osteoblast differentiation, and inhibits osteoclast formation [32]. A notable example is free fatty acid receptor 2 (FFAR2/GPR43), a SCFA-activated GPCR that helps coordinate metabolism and immune balance. Upon SCFA binding, GPR43 attenuates NF-κB signaling, reducing the production of lipopolysaccharide (LPS)-induced cytokines such as TNF-α and IFN-γ. This helps resolve inflammatory cascades and ultimately limits bone resorption [33,34,35]. Comparative studies in high-fat diet-induced obese (HIO) and non-obese (NO) mouse models showed that gut-derived SCFAs in NO mice dually enhance GPR43 signaling and inhibit histone deacetylase activity. This promotes regulatory T-cell (Treg) expansion and establishes an anti-osteoclastogenic microenvironment (Figure 2) [36].

One point worth clarifying is that SCFAs exhibit selective binding to GPR43 across different cell membranes. Current evidence indicates that butyrate mediates its effects indirectly—rather than engaging T-cell GPR43, it preferentially binds dendritic cell GPR43 through mechanisms independent of canonical GRP43 signaling [37]. Notably, recent studies highlight the therapeutic potential of targeting the SCFA–GPR41–p38 MAPK axis, which has shown promising effects against postmenopausal osteoporosis [38]. These findings underscore the importance of GPCR–SCFA interactions in advancing osteoporosis research and treatment.

It should be added that alternative mechanisms exist for SCFA-induced Treg cell expansion. Evidence indicates that the increase in Treg cell numbers following administration of SCFAs, such as butyrate, may also be attributed to enhanced de novo peripheral Treg differentiation [39]. This effect can be promoted by certain gut microbiota, such as Clostridium butyricum, which metabolizes acetate into butyrate [40,41]. However, some studies have observed different phenomena—despite elevated butyrate levels, Treg abundance did not increase significantly [42]. This suggests that the relationship between butyrate and Treg cells is not straightforward. A biphasic effect exists between butyrate and Treg cells: butyrate derived from Clostridium butyricum may establish a concentration gradient in the gut, reaching levels that locally suppress Treg function [43]. In contrast, Treg cells increased via probiotic pathways (e.g., *Lactobacillus rhamnosus*), together with SCFAs, can modulate WNT ligand expression, thereby activating the Wnt signaling pathway in osteoblasts to promote bone formation [44,45]. These findings provide important insights for developing interventions targeting osteoporosis (Table 1).

The dual role of butyrate suggests that the balance between SCFAs may regulate Treg cell dynamics. Butyrate has anti-inflammatory effects, while acetate enhances gut health and supports intestinal bone mineralization [46]. An optimal acetate-to-butyrate ratio seems essential. Since specific bacteria produce distinct SCFAs, microbial imbalances may lead to gut inflammation, though this hypothesis needs validation. Although SCFAs are key mediators in bone metabolism and current findings outline a framework for SCFA signaling, the translatability of animal model results to humans remains unclear.

In summary, SCFAs do not uniformly benefit osteoporosis. Treg cells, key mediators in this process, show a biphasic, concentration-dependent response to butyrate. The effect is further modulated by the gut microbiota composition and SCFA ratio, offering key insights into how microbial dysbiosis may lead to osteoporosis.

**Table 1:** Mechanisms of bone protection by different SCFAs.

SCFA Type	Representative Producing Microbes	Proposed Bone-Protective Mechanisms
Acetate	*Akkermansia muciniphila* *Bacteroides* spp. *Bifidobacterium* spp.	1. Maintains systemic metabolic homeostasis [47]. 2. Lowers intestinal pH, enhancing the solubility and absorption of minerals (e.g., calcium) [48]. 3. Activates GPR43 to indirectly inhibit bone resorption [49].
Propionate	*Bacteroides* spp. *Prevotella* spp. *Veillonella* spp.	1. Suppresses NF-κB signaling by activating GPCRs and inhibiting HDAC, reducing osteoclast differentiation [50]. 2. Promotes Treg expansion and inhibits pro-osteoclastogenic cytokines (e.g., TNF-α, IL-1β) [51].
Butyrate	*Faecalibacterium prausnitzii* *Eubacterium rectale* *Roseburia* spp.	1. Inhibits HDAC, leading to histone hyperacetylation-mediated suppression of osteoclastogenic genes (e.g., NFATc1) [52]. 2. Promotes osteoblast differentiation and mineralization through Wnt/β-catenin pathway activation [45]. 3. Maintains intestinal barrier integrity, thus reducing systemic inflammation from bacterial toxins [25].

Research on the osteoprotective effects of SCFAs reveals a complex endocrine network that mediates their regulation of bone homeostasis. Parathyroid hormone (PTH), the principal calcium-phosphorus regulator secreted by parathyroid chief cells, classically stimulates osteoclastogenesis to enhance bone resorption. It demonstrates synergistic effects with SCFAs in regulatory T cell (Treg) expansion while increasing osteoblast population [23,53]. Insulin-like growth factor-1 (IGF-1), a polypeptide hormone produced by the liver under growth hormone stimulation, is essential for growth and metabolic regulation. The gut microbiota directly modulates IGF-1 secretion, as shown by a marked increase in serum IGF-1 levels in germ-free (GF) mice after microbial colonization at both 1 and 8 months [54]. Remarkably, in antibiotic-treated models, SCFA supplementation not only restored IGF-1 concentrations but also brought bone mineral density back to baseline [29]. This strongly suggests that microbiota-derived SCFAs are key mediators of gut microbiota-induced IGF-1 elevation [54]. Elevated IGF-1 levels, in turn, promote the differentiation of osteoblasts, osteoclasts, and chondrocytes.

Meanwhile, SCFAs like propionate and butyrate activate specific receptors (e.g., GPCRs) on enterochromaffin cells [55]. This upregulates the rate-limiting enzyme TPH1 and stimulates the biosynthesis of gut-derived serotonin [56,57]. While SCFAs directly inhibit osteoclast differentiation and resorption, gut-derived serotonin enhances osteoclast activity [58]. This indicates that the skeletal effects of SCFAs involve a complex balance of synergistic and antagonistic interactions with gut-derived factors (e.g., serotonin, IGF-1) and specific hormones, although the precise regulatory pathways require further elucidation. These findings highlight that SCFAs are not isolated actors but integral parts of a complex biological network. The gut microbiota-mediated regulation of osteoporosis involves intricate interactions among multiple biochemical components, each contributing to the dynamic balance of bone homeostasis.

The interaction between SCFAs and gut microbiota exhibits close correlation with calcium-dependent mechanisms. Through direct structural remodeling of intestinal villi and functional expansion of the gut epithelium’s absorptive surface, SCFAs enhance the absorption and transport of calcium [59]. Butyrate specifically up-regulates the expression of intracellular calcium transporters, bringing about increased intracellular calcium absorption [60]. Current mechanistic understanding primarily attributes this process to the promotion of calcium absorption channel synthesis in intestinal epithelial cells, particularly calbindin-D9k (CaBP) and transient receptor potential vanilloid 6 (TRPV6) [61]. Calcium intake facilitates the adhesion of calcium-responsive microbiota, which are key producers of acetate and propionate (e.g., Acinetobacter and Propionibacterium) to intestinal epithelial surfaces [62]. This increases their abundance and SCFA production. The resulting SCFAs acidify the lumen, lowering pH and thereby promoting calcium ionization, solubility, and bioavailability [63]. Therefore, the observed positive bone axis effects can be substantially attributed to incremental SCFA biosynthesis [64].

Currently, there is a lack of direct clinical evidence on how SCFAs alleviate osteoporosis via endocrine pathways. Most available studies are intervention-based and rely on biomarkers (e.g., bone resorption markers) as endpoints, which provides only indirect evidence [65]. However, observational data cannot establish causality. Furthermore, butyrate’s bidirectional effects indicate that more SCFAs is not always better. Individual gut microbiota variations also lead to inconsistent responses to probiotics or prebiotics. Critical parameters—such as the effective SCFA dosage, preferred delivery route, and treatment duration needed to activate protective pathways in humans—are still unknown.

In conclusion, SCFAs function as indispensable signaling mediators, dynamically regulating cellular transduction cascades through receptor-coupled mechanisms. Future investigations should focus on exploring the expression of intestinal receptors, such as GPCRs, particularly at the genomic level, to unravel underlying molecular mechanisms. Such exploration could pioneer novel therapeutic strategies for osteoporosis through targeted genetic interventions in SCFA-mediated pathways.

### 2.2. Factors Associated with Inflammation Affecting the Modulation of SCFAs in Osteoporosis

#### 2.2.1. Probiotics, Prebiotics, and SCFAs Production

The abundance of SCFA-producing gut microbiota and their metabolite levels show strong positive correlations with increases in anti-inflammatory bacterial groups and immunoregulatory cytokines. This anti-inflammatory response constitutes a crucial mechanism through which SCFAs modulate osteoporosis. Various chemical and biological interventions targeting the skeletal system, including Tuna Bone Powder (TBP) [66], Gold Nanoparticles (GNPs) [67], and short-chain fructooligosaccharides (scFOS) [68], act by increasing SCFA levels and reducing inflammation to limit bone resorption.

Enrichment of anti-inflammatory and SCFAs-producing bacteria, along with elevated serum anti-inflammatory cytokines like IL-10, demonstrates considerable potential in countering osteoporosis through multiple biological pathways. Beyond SCFA production by anti-inflammatory bacteria, the enhanced colonic microbial metabolism stimulates the proliferation of fiber-fermenting obligate anaerobes that further amplify SCFAs generation [69]. The anti-inflammatory properties of SCFAs manifest through suppression of nuclear factor kappa-light-chain-enhancer of activated B cells (NF-κB), attenuating autoimmune inflammation [70]. Not only that, but oxidative stress represents another pathogenic contributor to osteoclast apoptosis. Multiple SCFAs derived from gut microbiota alleviate oxidative damage by stimulating antioxidant molecule production [71].

Additionally, the probiotic BL-99 can produce high levels of SCFAs by shaping the gut microbiota. It enriches Colidextribacter and upregulates goblet cell mucin MUC2, which strengthens the gut barrier and inhibits pro-inflammatory mediators. Therefore, appropriate probiotic intervention can be considered in the treatment of osteoporosis [72,73].

Emerging research suggests that SCFAs mitigate osteoporosis through two primary mechanisms. One is the direct anti-inflammatory improvement of the bone milieu, notably by elevating Tregs to suppress osteoclast formation and by restoring the RANKL/OPG balance, closely tying into osteoimmunology [28,74]. The other, examined in this review, operates through gut microbiota: SCFAs restore intestinal barrier integrity. Here, certain prebiotics and prebiotic-like compounds demonstrate significant potential. Puerarin counteracts osteoporosis by modulating gut microbiota, suppressing inflammation, and elevating SCFA levels to maintain intestinal homeostasis [75]. Fructooligosaccharide (FOS) or galactooligosaccharides (GOS) increase SCFA production, which reverses intestinal permeability and reduces inflammation, thereby protecting against HFD-induced osteopenia [76]. Similarly, Jian-Gu Granules restore gut microbiota diversity, enhance SCFA biosynthesis, and strengthen the colonic barrier, potentially via regulating the “Gut Microbiota-SCFAs-Treg/Th17 axis” [77].

In addition to SCFAs, other bioactive molecules like vitamin D and estrogen are involved in anti-inflammatory regulation against osteoporosis [65,78]. A healthy, SCFA-producing gut microbiota establishes an environment that enhances vitamin D absorption and estrogen metabolism. This environment, in turn, supports the complex immunomodulatory functions of vitamin D and estrogen, suggesting a potential anti-inflammatory synergy with SCFAs.

#### 2.2.2. Dietary Intervention and Environmental Factors

Mice fed high-fiber diets serve as a standard model for studying SCFAs’ relationship with osteoporosis. However, some high-fat diet (HFD) experiments contradict prior findings. For example, one study showed no significant protective effect of high-fiber diets on periodontal bone mass; alveolar bone loss was even higher in the high-fiber group [79]. Subsequent analysis revealed strong enrichment of inflammation-linked gut microbiota [80]. However, severely restricting fiber intake is not advisable. Existing animal studies have observed that excessive fiber restriction may lead to low-grade inflammation and metabolic issues in mice, such as growth retardation. These effects are long-lasting and not fully reversible, even with a subsequent high-fiber diet [81,82]. Thus, future studies should include dose–response experiments to accurately evaluate fiber’s therapeutic effects on osteoporosis and determine optimal fiber intake levels.

The influence of environmental factors on SCFA dynamics demands heightened scientific attention. Comparative studies show that low-altitude populations exhibit strikingly higher levels of acetic acid, butyric acid, and total levels of seven SCFAs compared to populations residing at higher altitudes. It strongly implicates altitude-induced metabolic adaptations as potential contributors to diminished BMD and elevated osteoporosis susceptibility [83]. SCFA levels also exhibit temperature dependence, with cold stress altering hepatic expression of genes related to bile acid and SCFA metabolism in animal models. However, the clinical relevance of these findings to human osteoporosis remains unconfirmed, necessitating comprehensive human studies [84].

These findings suggest multi-faceted interventions—dietary, pharmacological, and lifestyle—for bone health restoration, requiring tailored approaches based on individual metabolism. Large-scale studies are needed to establish clinical relevance. Targeting the gut–bone axis effectively requires usage guidelines, microbiota monitoring, and treatment evaluation. Understanding the underlying mechanisms will improve precision and reduce side effects.

Notwithstanding significant advances, critical knowledge gaps persist in the study of dietary and environmental influences. Future research should focus on: (1) establishing tolerated doses and health-benefit thresholds for dietary fiber across diverse populations; (2) elucidating the mechanisms underlying the impact of high-altitude environments on SCFA metabolism; and (3) translating animal-based findings on the temperature dependence of SCFA levels into clinical evidence in humans.

### 2.3. Potential Approaches to Relieve Osteoporosis via SCFAs

Based on the established link between SCFAs and bone mineral density, this review outlines potential therapeutic strategies (see Table 2). Strategically modulating gut microbiota to influence SCFA biosynthesis allows for bone mass regulation by targeting osteogenic enzyme activity and osteoblast proliferation. This approach has emerged as a key osteoporosis intervention, where SCFAs’ antioxidant properties help maintain intestinal equilibrium.

Growing research focuses on SCFAs’ role in regulating calcium absorption, aiming to address osteoporosis via a controlled calcium–SCFA balance. Advanced biomaterial engineering enhances SCFA delivery and stability to boost therapeutic efficacy. Dietary intervention remains a cost-effective frontline approach, with numerous studies promoting high-fiber, plant-based polysaccharides to elevate SCFA levels. Future research should prioritize gut microbiota balance, offering novel perspectives for treating skeletal disorders.

Important knowledge gaps persist regarding: (1) optimal pre/probiotic doses; (2) long-term efficacy of SCFA interventions; and (3) mechanistic interactions between SCFAs, vitamin D, and estrogen. Although SCFAs—important gut microbiota metabolites—contribute to osteoporosis, their mechanism is primarily indirect via anti-inflammatory pathways, and robust clinical evidence is still lacking. Existing studies, which often evaluate probiotics as PMO adjuncts or track SCFA changes after intervention, are limited by uncontrolled confounders like diet and exercise [65,85].

Moving forward, research should investigate combination strategies—diet, probiotics, and advanced delivery systems—and personalize SCFA modulation based on individual microbiomes. This requires addressing the risks of SCFA over-supplementation, high interpersonal variability, and long-term adherence challenges.

**Table 2:** Potential therapeutic strategies utilizing SCFAs for osteoporosis treatment.

Category	Treatment Method	Microbiota Targeted	SCFAs Produced	Mechanism
Biomaterials/Engineered Nanoparticles	*Spirulina platensis* (SP)	*Turicibacter* Firmicutes Bacteroidetes	Propionate Butyrate	Reduces oxidative stress, enhances Wnt signaling, and suppresses osteoclast formation, significantly improving bone mineral density (BMD) [86].
	Sheep bone protein Hydrolysate	Thick-walled Bacteria Proteobacteria Verrucomicrobia	Propionate Butyrate	(1) Modulates gut microbiota composition and enhances intestinal calcium uptake. (2) Boosts osteoblast numbers and stimulates bone regeneration by regulating both gut microbiota and osteogenic activity [59].
	Β-TCP/P (3 HB) bracket	—	3-Hydroxybutyric acid	Converts 3-hydroxybutyrate to 3-hydroxybutyric acid, supporting tissue health and reducing osteoporosis [87].
	Colon-targeted engineered postbiotics nanoparticles	*Shigella dysenteriae* Alistipes	Butyrate	(1) Allows targeted colon delivery and controlled butyrate release, avoiding toxicity from sudden concentration spikes. (2) Restores gut balance by regulating redox status, macrophage behavior, and microbiota structure [88].
Prebiotics	Inulin	Allobaculum Bifidobacterium	Acetate Propionate Butyrate	(1) Provides energy for microbial metabolism in the gut and acidifies the intestinal environment. (2) Activates TRPV6 calcium channels and enhances AQP8-mediated water flux, synergistically promoting passive calcium absorption [64].
	*Cistanche deserticola* Polysaccharide (CDPS)	Butyrate-producing bacteria (e.g., *Lachnospiraceae* NK4A136 group)	Butyrate	Suppresses overactivation of the SRC/EGFR/PI3K/AKT signaling axis [89].
	*Lycium barbarum* polysaccharide (LBP)	Sclerobacillus Lactobacillus Turicibacter Clostridium_sensu_stricto_1 Faecalibacterium Adlercreutzia	Acetate Propionate Butyrate	Upregulates alkaline phosphatase (ALP) * biosynthesis and enzymatic activity, promoting osteoblast differentiation and maturation [90].
	Fructooligosaccharide (FOS)	Bifidobacterium	Butyrate	Enhances peak bone mass (PBM) and prevents estrogen deficiency-induced bone loss by selectively stimulating new bone formation [68].
Diet	Green tea	Akkermansia	Butyrate	Significantly modulates gut microbiota, enhances intestinal antioxidant capacity, and regulates bone metabolism [91,92].
	Calcium-fortified diets	*Acinetobacter* *Propionibacterium*	Acetate, Propionate	Increases luminal soluble/available calcium and stimulates expression of calcium absorption-related genes, ultimately improving bone mineral density (BMD), bone mineral content (BMC), and femoral mechanical strength [64].
	Mediterranean diet	*Bacteroidetes* *Thick-walled Bacteria*	Propionate Butyrate	Modulates specific gut microbiota associated with osteoclast suppression and promotes SCFA production [93].
	Vegetarian Diet	Bacteroides Prevotella	Acetate Propionate Butyrate	Reduces bone metabolic disorders, provided adequate intake of calcium, vitamin D, and protein is maintained [9].

* ALP, a critical protein involved in bone metabolism and an early marker of osteoblast maturation, reflects the degree of osteoblast differentiation through its activity.

## 3. Estrogen

### 3.1. The Role of Estrogen in Intestinal Metabolism

#### 3.1.1. Lipid Metabolism

**Figure 3:**
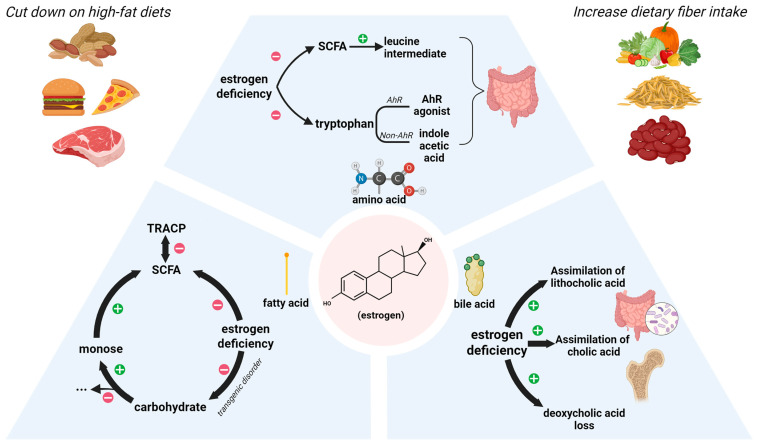
Estrogen mitigating osteoporosis via intestinal metabolism. Created in BioRender. Liang, Xinping (2025), https://BioRender.com/ynwhh8r (accessed on 2 June 2025).

In investigating the role of SCFAs in osteoporosis, many researchers have noted that estrogen demonstrates significant metabolic correlations with the aforementioned SCFAs. Estrogen deficiency stands as one of the primary etiological factors underlying postmenopausal osteoporosis. Such deficiency leads to diminished populations of butyrate-producing gut microbiota, including *Clostridium leptum*, *Clostridium coccoides*, *Faecalibacterium prausnitzii*, and *Roseburia* [94,95], which manifests profound consequences in bone loss. Current mechanistic investigations suggest potential associations with transcriptional dysregulation (Figure 3).

Estrogen-deficient OVX mice display induced transcriptional dysregulation specifically linked to lipid metabolism [96]. This change suppresses certain carbohydrate pathways, increasing the breakdown of complex carbs into monosaccharides. These are then fermented by gut microbes to produce butyrate and other SCFAs [97]. It is a process attenuated under estrogen-deficient conditions. Concurrently, estrogen exerts regulatory control over the concentration of TRACP, a crucial biomarker of bone resorption that normally shows significant elevation post-ovariectomy. Notably, this investigation revealed a remarkable association between 2-hydroxybutyrate and TRACP levels [98]. These findings collectively demonstrate that estrogen-mediated regulation of SCFAs can be quantitatively reflected through such biomarker profiling. However, estradiol’s influence on gut microbes and SCFAs also depends on exposure time. Different treatment durations shift microbial communities along the gut, changing metabolite levels [99].

Through SCFA-producing bacteria, estrogen affects lipid metabolism and bone remodeling, which can be monitored using bone resorption markers. Still, estrogen’s impact on lipid pathways depends on multiple factors, including gut environment and exposure duration.

#### 3.1.2. Amino Acid Metabolism

Studies show that estrogen (E2) regulates not only fatty acid metabolism, but also amino acid and bile acid metabolism [100]. For instance, butyrate metabolism generates leucine intermediates, with estrogen demonstrating the capacity to regulate intestinal morphology through leucine modulation via this pathway [75].

Furthermore, treatment with E2 (17β-estradiol), a predominant form of estrogen, has been shown to regulate multiple amino acids encompassing lysine, alanine, tryptophan, and asparagine [100]. Tryptophan, essential for protein synthesis and a precursor to many bioactive molecules, is involved in multiple metabolic pathways. Recent research indicates that tryptophan helps restore gut barrier integrity and reduce bone loss in ovariectomized mice, acting through the AHR pathway [101]. Beyond the classical AhR pathway, evidence suggests that the tryptophan metabolite indoleacetic acid promotes estrogen production via gut bacteria such as *Bifidobacterium pseudolongum*, supporting intestinal homeostasis [102]. This reaffirms the existence of positive feedback regulation and synergistic interactions between gut microbiota and biological signaling molecules.

Due to its regulatory role in amino acid pathways, estrogen’s protective effect on the intestinal barrier is further amplified. This helps reduce inflammatory infections in the gut and creates a relatively stable environment for the gut microbiota. As a result, this decreases disruptions to bone metabolism involving osteoblasts and osteoclasts.

#### 3.1.3. Bile Acid Metabolism

Cholic acid, synthesized from hepatic cholesterol, constitutes one of the primary bile acids. It facilitates the digestion and absorption of fat-soluble vitamins, including vitamin D. In the gut, some cholic acid is converted by gut bacteria into secondary bile acids—including deoxycholic acid and lithocholic acid—which help regulate bone metabolism [12,103]. Ovariectomy paradoxically enhances cholic acid and lithocholic acid assimilation while accelerating deoxycholic acid depletion [100]. Recent studies identify lithocholic acid as a key metabolite in disuse-induced osteoporosis (DIO) mice, where bile acid metabolism becomes abnormally active [104]. Given the potent membrane-disruptive properties of bile acids against microbial communities, researchers propose that regulating dietary fat intake could effectively suppress bile acid synthesis. Generally speaking, bile acids impose substantial ecological pressure on gut microbiota, potentially destabilizing intestinal homeostasis [105].

Research indicates that bile acid metabolism’s impact on the gut–bone axis cannot be oversimplified. Studies confirm a robust link between the bile acid receptor TGR5 and gut microbiota depletion triggered by estrogen deficiency [106,107]. Intriguingly, microbiota depletion exerts divergent effects across life stages: During non-menopausal periods, it reduces bone mass [108], while antibiotic-induced depletion after menopause paradoxically increases bone mass and mineral content [109]. Though evidence for TGR5′s role in the former mechanism remains incomplete, this underscores the necessity of examining distinct life stages—particularly menopausal versus non-menopausal periods—in future GBA research. Crucially, menopause represents the most critical phase of bone loss in women. Prioritizing separate analysis of this stage, with comparisons to other periods, is imperative for advancing current studies.

It is noteworthy that long-term use of estrogen supplements may carry potential adverse effects. Prolonged estrogen administration increases cholesterol saturation in bile, suppresses the function of canalicular transporters, and elevates the risk of cholestasis and gallstone formation [110]. Additionally, it disrupts the gut microbiota composition, alters the ratio of primary to secondary bile acids, and reduces the production of beneficial short-chain fatty acids such as butyrate [106,111].

Bile acid metabolism is a key pathway mediating estrogen’s influence on bone mass. Given the dual role of estrogen in this process, further investigation into the dosage, timing, and duration of treatment is essential. These insights advance our understanding of the gut–bone axis.

#### 3.1.4. Practical Therapeutic Implications and Clinical Relevance

Common environmental and dietary factors can significantly disrupt gut metabolism and estrogen signaling. For example, polycyclic aromatic hydrocarbons (PAHs), produced during fossil fuel combustion and food grilling, act as ligands for the aryl hydrocarbon receptor (AhR). These compounds interfere with amino acid metabolism, induce gut inflammation, alter gut microbiota, and impair estrogen receptor sensitivity [112,113]. Similarly, inhalable particulate matter (PM2.5/PM10) from air pollution can reduce gut microbial diversity and deplete beneficial bacteria that generate SCFAs [114,115].

Environmental endocrine-disrupting chemicals (EDCs), often called “environmental hormones,” are exogenous compounds that disrupt the normal function of the endocrine system in organisms. Studies have shown that EDCs such as zearalenone (ZEA), which binds to estrogen receptors, can modulate glucose homeostasis and lipid metabolism by altering the gut microbiota. This evidence highlights the important role of estrogenic pathways in metabolic regulation [116]. For metabolic disorders caused by gut dysbiosis, targeting estrogen receptors offers a promising therapeutic approach. For example, selenium nanoparticle supplementation has been found to protect estrogen receptor (ER) function, counteracting ZEA-induced gut microbiota imbalances and increasing the abundance of short-chain fatty acid-producing bacteria such as Alloprevotella and Muribaculaceae [117].

The impact of dietary supplementation on gut metabolism in estrogen-deficient individuals warrants further attention. While most previous studies have examined gut metabolism in conventional ovariectomized mouse models, recent work, though still limited in clinical evidence, has advanced our understanding. For instance, intake of fiber- and polyphenol-rich foods like black currants and dried plums has been shown to reshape the gut microbiota in postmenopausal women [118,119]. This remodeling promotes microbial fermentation of dietary fiber, boosts SCFA production, and ultimately benefits bone health.

It is also important to note that a high-fat diet, while stimulating the growth of β-glucuronidase-rich bacteria and enhancing estrogen reabsorption, can simultaneously alter bile acid metabolism and interfere with estrogen signaling [120,121]. Thus, high-fat diets are generally recommended to be restricted.

Prebiotics selectively promote the growth and activity of beneficial gut bacteria (e.g., *Bifidobacterium*, *Lactobacillus*) in postmenopausal women. They also modulate bile acid metabolism in individuals with bone loss, reducing pro-inflammatory secondary bile acids and thereby preserving intestinal barrier integrity [122,123]. Furthermore, bacterial fermentation of prebiotics like fructooligosaccharides (FOS) and inulin produces SCFAs, including propionate and butyrate [124].

Current clinical evidence is limited due to confounding factors like physical activity and environment. Most data come from OVX animal models, highlighting key constraints of preclinical systems: (1) Gut microbiota differ significantly between mice and humans at multiple taxonomic levels. Bacterial changes in mice may not occur or function the same way in humans. (2) Physiological and immune differences in the gastrointestinal tract affect the absorption and distribution of drugs, nutrients, and microbial metabolites. Thus, effective doses and metabolic pathways in mice may not translate directly to humans. (3) Surgically induced menopause in mice is acute, unlike the gradual estrogen decline in humans. This limits the model’s ability to replicate the chronic, complex pathophysiology of postmenopausal osteoporosis (PMO).

These limitations underscore the need for more representative preclinical models in future studies.

### 3.2. Research Progress on Estrogen–Gut Microbiota Interactions and Associated Limitations

The interplay between estrogen and gut microbiota has operated through a dynamic homeostatic equilibrium. Anna Clapp Organskia et al. demonstrated through conventional housing and gnotobiotic mouse models that transplantation of gut microbiomes from sex hormone-modified mice into germ-free recipients could disrupt hypothalamic–pituitary–gonadal (HPG) axis regulation [125]. A one-year randomized, double-blind, placebo-controlled trial further demonstrated that supplementing osteoporotic women with phytoestrogens, such as standardized 8-PN hop extract, can alter gut microbiota abundance and consequently increase total-body BMD [65]. These findings collectively establish a reciprocal regulatory dialogue between gut microbiota and estrogen, rather than unidirectional influence. It is characterized by complex yet stable interactions.

Estrogen’s role in substance metabolism manifests through its ability to drive critical microbial community changes associated with bile acid, amino acid, and fatty acid metabolism [100]. In fact, in OVX mice, this remodeling suppresses dominant microbial groups and enriches subdominant ones, significantly altering community structure. This is exemplified by a marked increase in the Firmicutes/Bacteroidetes ratio [126], with several key genera within these phyla showing correlations with serum biomarkers of bone homeostasis, particularly TNF-α and IL-17 [66]. Similarly, in postmenopausal osteoporotic women, estrogen deficiency leads to a depletion of bacteria involved in the tryptophan-indole pathway, such as the BMD-positive Bacteroides [127].

A mutual regulatory mechanism exists between estrogen and gut microbiota. Estrogen not only modulates microbial composition but also maintains the intestinal barrier integrity mediated by gut microbiota. As a key regulator of the intestinal barrier, estrogen enhances barrier function by upregulating tight junction proteins via estrogen receptor b signaling [128], or by increasing mucin gene expression to reinforce physical segregation between the host and microbiota [129]. Additionally, estrogen exerts indirect regulatory effects on the intestinal barrier via microbiota modulation [130]. For instance, gut microbes release metabolites such as short-chain fatty acids and bile acids into the gut lumen, which dynamically regulate barrier permeability [131]. Estrogen deficiency disrupts the equilibrium, amplifying intestinal permeability to microbial metabolites and compromising barrier selectivity [132].

In turn, intestinal flora can also regulate the secretion of estrogen. Recent scientific literature has introduced the concept of the ‘estrobolome’, a collection of microbial genetic elements that directly govern the biosynthesis and catabolism of sex steroids, including estrogen, through specialized metabolic networks [133]. This discovery provides compelling evidence for the regulatory capacity of gut microbiota in modulating systemic estrogen levels. A host of surveys have shown that the regulation is in connection with particular metabolic pathways. A recent study revealed that *Lactobacillus plantarum* FRT 4 can elevate estrogen levels by remodeling gut microbiota composition and function, thereby intervening in the FoxO/TLR4/NF-κB signaling pathway to exert antioxidant and anti-inflammatory effects [134]. However, these findings are currently limited to animal models and await validation in humans.

Accumulated clinical studies show that gut dysbiosis alters metabolic profiles, damages the intestinal mucosal barrier, and leads to L-glycosylpurine accumulation. By inhibiting AMPKα/MFF-driven mitochondrial fission, L-glycosylpurine disrupts mitochondrial homeostasis and ultimately suppresses estradiol production [135], revealing a novel perspective on postmenopausal osteoporosis.

Recent studies have increasingly investigated bone loss in perimenopausal and postmenopausal women. Marked by estrogen deficiency, these individuals exhibit more pronounced alterations in gut microbiota—such as α- and β-diversity and fungal composition—when compared to non-menopausal osteoporosis groups [136]. Moreover, changes in the abundance of specific bacterial taxa are significantly associated with vitamin K production by particular intestinal bacteria [137,138].

An assessment of trends over the past 30 years indicates that postmenopausal women are at a higher risk for bone loss and related fractures than premenopausal women [139]. Metagenomic analysis reveals that the gut microbiota of postmenopausal women converges toward a male-like profile, a shift associated with low estrogen and progesterone levels. In contrast, the gut microbiome of premenopausal women is enriched in genes for steroid biosynthesis and degradation, pathways significantly correlated with progesterone levels [140].

Prior to menopause, high and fluctuating estrogen levels are associated with a relatively stable and diverse gut microbiota [141]. In contrast, the onset of menopause and the subsequent sharp decline in estrogen disrupt this homeostasis, leading to substantial structural shifts and reduced microbial diversity [126]. Crucially, premenopause is characterized by a dynamic bidirectional balance: estrogen shapes the microbiota, which in turn modulates estrogen recycling via β-glucuronidase [142]. After menopause, however, diminished estrogen substrate and microbial imbalance jointly suppress β-glucuronidase activity, breaking this balance [120,138]. Consequently, gut microbiota structure and diversity differ significantly before and after menopause.

While hormone replacement therapy (HRT) has been a conventional approach for PMO, its clinical utility is constrained by breast carcinogenesis and cardiovascular risks [143,144]. Microbiota modulation may present a superior alternative to HRT, though more rigorous long-term clinical trials are imperative to minimize potential risks and validate therapeutic efficacy. Estrogen supplementation exhibits a plateau effect regarding bone health benefits: beyond a certain point, additional dosage provides no extra advantage. Identifying the optimal therapeutic dose is therefore essential.

Research exploring sex hormone-gut microbiota interactions, particularly in randomized clinical trials, reveals that participants frequently demonstrate some degree of vitamin D deficiency [145]. This deficiency may influence study outcomes, as estrogen supplementation exerts differential effects in vitamin D-insufficient versus sufficient individuals [65]. Consequently, a comprehensive vitamin D status evaluation is recommended before initiating estrogen therapy.

Although research on gut microbiota and estrogen continues to advance, many questions remain unresolved. First, due to inherent genetic heterogeneity among subjects, individual variations in microbiota can influence estrogen metabolism [146]. When addressing osteoporosis, the choice between microbiota-targeted interventions and HRT warrants careful consideration. Probiotic supplementation acts slowly, requires long-term use, and exhibits strain-specific and individually variable effects [147]. However, its reported side effects are generally mild and transient [148]. In contrast, HRT is more suitable for relieving moderate-to-severe menopausal symptoms—it acts rapidly but carries established risks [149]. These issues call for deeper investigation in future studies on osteoporosis.

### 3.3. ERα/β and Metabolite Receptor and Gut Microbiota Dysbiosis-Induced Osteoporosis

Estrogen deficiency-induced gut microbiota depletion contributes to osteoporosis development, with specific receptor-ligand interactions playing crucial roles. As established in previous research, gut microbiota alterations influence bile acid metabolism, particularly through modulation of bile acid receptor TGR5 [150]. TGR5 demonstrates dual protective mechanisms by suppressing osteoclast differentiation to prevent estrogen-dependent bone loss in mice, while its activation simultaneously enhances osteoblast formation [106].

Estrogen exerts its biological effects primarily through classical estrogen receptors ERα and ERβ. Intriguingly, although ERβ shows predominant expression in estrogen-sensitive tissues like bone [151], ERα retains substantial osteogenic potential through synergistic activation with the Wnt/β-catenin signaling pathway [152]. This suggests ERα’s potential involvement in postmenopausal osteoporosis pathogenesis. ERβ, which mainly exists in bone and other tissues, is capable of binding to estrogenic compounds, including isoflavones and equol, triggering the ERβ-induced cell signaling pathway. This ligand-receptor interaction causes changes in the expression of cellular enzymes (such as superoxide dismutase, catalase, etc.). Through these molecular mechanisms, ERβ mediates estrogen’s antioxidant effects, effectively mitigating oxidative stress-induced bone deterioration caused by reactive oxygen species and related cytotoxic agents [153,154]. It is worth noting that ERβ exhibits the remarkable capacity to modulate the gut microbial composition. This microbial restructuring effectively suppresses the pathogenesis of inflammatory bowel disease while concurrently emerging as a promising therapeutic strategy for mitigating osteoporosis progression [155].

Phytoestrogens are frequently employed in gut–bone axis research to mimic endogenous estrogen. However, their physiological outcomes differ from those of human estrogen in vivo. Soy isoflavones, for example, exhibit only 1/1000th the binding affinity for ER compared to endogenous estrogen. Critically, their reliance on gut microbiota for metabolic activation renders their effects heavily dependent on microbial composition. This dependence may mask functions of endogenous estrogen while overstating microbiota’s role. Thus, future studies should design more rigorously controlled experiments.

Beyond phytoestrogens, potential interventions targeting ERα/β or TGR5 are under investigation. Raloxifene is currently the only SERM approved for postmenopausal osteoporosis, acting as an estrogen agonist in bone to inhibit resorption and increase density [156]. Although no TGR5-targeting drugs have reached clinical use, certain agonists like betulinic acid derivative SH-479 have been shown to reduce bone loss in mice [157]. Additionally, TGR5 plays a key role in the anti-osteoporotic effects of other substances, such as the iridoid glycoside Specnuezhenide (SPN) and folic acid [107,158]. However, evidence for TGR5 agonists remains confined to animal models, warranting further exploration in human physiology.

Growing knowledge of estrogen receptors still leaves important questions unresolved. These include the undefined roles of ERα and ERβ in the gut, how gut microbiota diversity influences phytoestrogen metabolism, and the lack of systematic sex-based comparisons in animal studies of ERβ and TGR5. Current evidence is also centered on postmenopausal women, raising the question of whether results apply equally to men.

In brief, microbial metabolites like SCFAs and phytoestrogens act as key communicators between the gut and bone. Their activation of specific gut receptors—GPR41/GPR43 for SCFAs and ERβ for phytoestrogens—modulates immunity and gut barrier integrity, ultimately influencing bone metabolism. Despite growing insight into gut microbiota dynamics in postmenopausal osteoporosis, the precise mechanisms are not yet fully understood. Deeper molecular and pathway analyses will clarify this bidirectional interaction. Importantly, gut microbiota profiling may help define clinical subtypes of the disease, paving the way for improved future detection.

## 4. Vitamin D

As a nuclear transcription factor integral to bone metabolism, vitamin D exerts indispensable regulatory functions in skeletal health maintenance. The scientific community recognizes that vitamin D, through its bioactive metabolite 1,25-dihydroxyvitamin D3 (1,25(OH)_2_D), is instrumental in calcium homeostasis regulation and orchestrates anti-inflammatory mechanisms via the IL-33/ST2 axis, thereby profoundly influencing bone metabolism [159]. During investigations into the pathogenesis of postmenopausal osteoporosis, researchers have identified a novel pathway designated as “*C. maltaromaticum*/7-DHC/*F. prausnitzii*/Vitamin D3”. This pathway elucidates how estrogen stimulation selectively enriches *Lactobacillus maltaromaticum* populations within gut microbiota and facilitates vitamin D3 biosynthesis [160]. The interplay between vitamin D3 and gut microbiota has consequently garnered significant scientific attention. Recent empirical evidence has shown that vitamin d has relevance to maintaining intestinal mucosal barrier integrity, serving as a pivotal regulator in balancing intestinal homeostasis and osteoporosis development.

### 4.1. The Symphonic Interplay Between Vitamin D Signaling and Intestinal Mechanical Barrier Homeostasis

The intestinal mucosal barrier constitutes a tripartite system comprising: (1) a physical barrier formed by intestinal epithelial cells, (2) a chemical barrier involving vitamin D and related components, and (3) a biological barrier established by gut microbiota. Compromise of the frontline physical barrier, manifesting as villous atrophy or mucosal damage, impairs vitamin D bioavailability and calcium absorption, consequently causing vitamin D deficiency [161].

Conversely, vitamin D exerts reciprocal regulatory effects on intestinal mucosal integrity. Gubatan, J. et al. demonstrated that vitamin D/VDR signaling modulates the expression and functionality of tight junction proteins, particularly maintaining Occludin and E-cadherin protein levels while inhibiting enterocyte apoptosis [162,163]. Accordingly, VDR knockout or vitamin D-deficient models display disrupted tight junctions, increased gut permeability, and impaired barrier function. This breakdown in intestinal homeostasis promotes pathogenic bacterial colonization and enterotoxin release, creating a vicious cycle of barrier deterioration [164,165,166,167]. Furthermore, vitamin D indirectly affects tight junctions by modulating intestinal calcium uptake. Calcium is a key regulator of keratinocyte differentiation and cornification, involved in signaling pathways that control desmosome and tight junction formation [159].

Existing evidence illuminates the intricate interplay between vitamin D and the gut microbiota-bone axis through multifaceted anti-inflammatory pathways. Experimental models demonstrate that transgenic mice with VDR overexpression exhibit suppressed intestinal epithelial inflammation, while adequate vitamin D status correlates with marked attenuation of skeletal system inflammatory responses. This immunomodulatory effect appears mediated through enhanced secretion of antimicrobial peptides and immunoregulatory molecules, particularly interleukin-10 (IL-10) [159,167]. Additionally, VDR upregulates occludin in tight junctions, while vitamin D suppresses levels of claudin [168,169].

Notably, vitamin D contributes to anti-inflammation by promoting the growth of beneficial, SCFA-producing bacteria, including *Bifidobacterium* and *Lactobacillus* [170,171]. Beyond this, the vitamin D/VDR pathway enhances the expression of enzymes responsible for bile acid detoxification in the gut. This helps convert toxic bile acids like LCA into less toxic forms, thereby protecting the intestinal mucosa [172,173].

Collectively, these findings underscore that vitamin D supplementation holds dual therapeutic potential for fortifying intestinal barrier integrity and ameliorating osteoporotic progression.

While mechanistic evidence from mice is strong, human clinical data remain limited. Most trials focus on vitamin D’s effects on bone density and metabolism, rarely assessing gut barrier integrity directly via biomarkers. Furthermore, evidence for its gut barrier benefits relies largely on indirect observations, such as microbiota modulation and reduced systemic inflammation, effects which are highly variable.

Recent trials combining vitamin D with pro- and prebiotics improved bone markers in at-risk postmenopausal women, sometimes replacing medications [174]. More studies directly linking vitamin D to gut barrier function are needed, though they must account for population heterogeneity.

### 4.2. Interaction Between Vitamin D and Intestinal Microbiota

**Figure 4:**
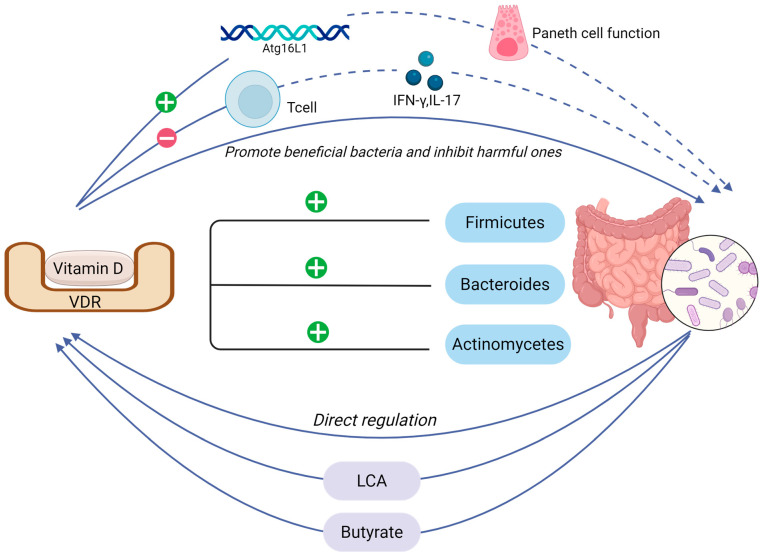
Mechanism of vitamin d affecting intestinal tract. Created in BioRender. Liang, Xinping (2025), https://BioRender.com/khvp6jb (accessed on 2 June 2025).

Beyond enhancing intestinal epithelial cell responsiveness, increased vitamin D may also regulate gut microbes that act as a biological barrier within the mucosal barrier. There was evidence that participants with optimal 25(OH)D concentrations exhibit significantly higher gut microbiota diversity compared to those with vitamin D insufficiency (*p* < 0.05) [175]. Vitamin D levels modulate both the abundance and metabolic properties of specific commensal species exhibiting vitamin D-dependent ecological niches, notably Bacteroides fragilis [176]. Intervention studies reveal vitamin D supplementation elevates bacterial abundance at both phylum and genus levels: Firmicutes, Bacteroidetes, and Actinobacteria at the phylum level; Faecalibacterium, Ruminococcaceae, Coprococcus, and Akkermansia at the genus level (Figure 4) [177,178].

The balance between effector T cells (Th1/Th17) and regulatory T cells is key to how vitamin D shapes the gut microbiome [179]. Vitamin D can inhibit T cells from releasing IFN-γ and IL-17 and induce T cells in mucosal tissues to regulate intestinal microbes. Furthermore, vitamin D directly stimulates proliferation of beneficial bacteria while inhibiting pathogenic overgrowth, enhancing intestinal barrier function and attenuating subsequent immune hyperactivation [180]. High-dose vitamin D3 supplementation selectively diminishes opportunistic pathogens (*Pseudomonas*, *Escherichia*, and *Shigella* spp.), creating niche opportunities for commensal expansion and increased microbial diversity [181]. This microbiota remodeling directly upregulates colonic epithelial vitamin D receptor expression, establishing a VDR-mediated negative feedback loop that counteracts NF-κB activation induced by microbial components [182].

The role of anti-inflammatory factors in VDR and Treg activity is equally significant. TGF-β drives Treg differentiation, and VDR acts in concert with TGF-β signaling to promote immune tolerance [183,184]. Subsequently, VDR-driven Tregs increase IL-10 secretion [185]. Ultimately, VDR, Tregs, and anti-inflammatory cytokines work together to exert anti-inflammatory effects and preserve the intestinal environment.

Aside from the identified contributing factors, the potential relationship between intestinal mechanical barrier integrity and microbial colonization dynamics warrants further investigation. Specifically, whether structural features such as well-maintained tight junctions and reduced epithelial cell apoptosis create an accelerated colonization timeline and a favorable growth environment for gut microbiota proliferation remains inadequately supported by current evidence. It needs to be further verified.

Vitamin D and gut microbiota collectively make up a vast and intricately interconnected micro-ecosystem. A reciprocal relationship exists between vitamin D and microbial communities, with gut microbiota playing an indispensable role in modulating intestinal vitamin D metabolism and its systemic circulation. Bora SA et al. demonstrated that germ-free mice infected with pathogen *C. rodentium* exhibited impaired serum absorption of both 25-hydroxyvitamin D and 1,25-dihydroxyvitamin D [186]. Subsequent studies confirm that probiotics like *Lactobacillus plantarum* and *Lactobacillus reuteri* boost VDR protein expression [187], demonstrating that the gut microbiota regulates VDR despite not expressing it.

As previously discussed, lithocholic acid (LCA), a key gut bacterial metabolite derived from primary bile acids by 7α-dehydroxylase-expressing bacteria (e.g., *Clostridium scindens*), contributes to osteoporosis pathogenesis via host-microbiome interactions [188,189]. VDR is a key receptor for LCA [190], and gut microbes enhance vitamin D bioavailability through this bile acid [191]. Notably, vitamin D and bile acid metabolism are linked by a shared nuclear receptor network involving VDR, farnesoid X receptor (FXR), and pregnane X receptor (PXR) [192]. VDR and PXR both bind bile acids and vitamin D, whereas FXR activation regulates active vitamin D levels by controlling vitamin D hydroxylase activity, forming a bidirectional regulatory circuit [193,194]. Together, these receptors jointly regulate lipid absorption and microbial composition.

Consistent with the above findings, the dynamic interplay among vitamin D, its receptors, and butyrate-producing bacteria deserves further investigation. Butyrate enhances intestinal VDR expression and mitigates inflammatory responses, thereby reinforcing intestinal mucosal integrity [195]. Additionally, VDR’s regulatory effects on SCFAs have garnered significant attention. Experimental evidence indicates that males with higher 1,25(OH)2D levels and activation ratios demonstrate a greater abundance of butyrate-producing bacteria [196]. Vitamin D receptors have the capacity to modulate the production of SCFAs like butyrate through alterations in gut microbial homeostasis, subsequently initiating a cascade of regulatory effects on both gut microbiota and bone metabolism [197]. Illustrating this paradigm, vitamin D deficiency impairs Atg16L1-mediated autophagy in Paneth cells, causing a severe depletion of butyrate-producing bacteria [198]. This observation aligns with the established role of VDR in microbial regulation. Furthermore, associations between vitamin D and certain polyunsaturated fatty acids (e.g., dietary omega-3) have been reported [199], which may influence osteoporosis pathogenesis through direct or indirect modulation of the gut microbiota.

Beyond secondary bile acids and short-chain fatty acids, other microbial metabolites, including polyamines, also regulate vitamin D metabolism. As key compounds derived from gut microbiota, polyamines enhance VDR signaling by stabilizing the vitamin D receptor protein [200].

It is noticeable that some non-Asian studies have yielded contradictory findings. A recent Australian trial involving healthy participants aged 60–84 demonstrated no significant effect of vitamin D supplementation on the abundance of different bacterial genera (mean values: 3.51 vs. 3.52; *p* = 0.50) [201]. These discrepancies may arise from variations in study design and population demographics. Since factors like sex hormones and age influence both vitamin D metabolism and gut microbiota, overlooking these confounders can easily lead to incorrect conclusions [202,203].

The method of vitamin D supplementation is determined by the form administered. While 25(OH)D has little direct effect on the gut microbiota, its levels are crucial as it is the precursor to the active metabolite, 1,25(OH)2D. This hormonally active form potently regulates the composition and function of the gut microbiota.

The significant influence of diet and geography on gut microbiota must be acknowledged. Elucidating these complex interactions requires large-scale randomized controlled trials. Developing personalized interventions for populations with diverse geographic, lifestyle, and vitamin D status is crucial.

Through its effects on gut microbiota diversity, intestinal barrier integrity, and anti-inflammatory immune responses (e.g., Treg promotion and Th1/Th17 suppression), Vitamin D collectively establishes a microbiota-immune axis relevant to bone health. This pathway offers a potential strategy for preventing osteoporosis, though its efficacy depends on individual variation, vitamin D form, and environmental context, highlighting the need for personalized approaches.

### 4.3. Vitamin D-Driven Recombination of Intestinal Flora for Osteoporosis Treatment

The intricate synergy between vitamin D and gut microbiota opens new therapeutic horizons for osteoporosis management. Potential interventions range from direct vitamin D supplementation to indirect nutritional approaches such as omega-3-enriched dietary regimens to maintain gut microbial equilibrium. Ultraviolet (UV) phototherapy has emerged as another viable option. UVB exposure increases serum vitamin D levels and enhances microbial diversity. It also promotes the activation of intestinal immune cells, leading to beneficial microbial restructuring and improved skeletal metabolism through increased osteogenic signaling [204,205,206]. Vitamin D synthesis from sunlight varies with latitude and lifestyle. In high-latitude regions, insufficient UVB exposure reduces vitamin D production, potentially causing microbiota dysbiosis and diversity loss. In contrast, lower latitudes are associated with richer gut microbiota diversity. Likewise, frequent outdoor activity correlates with higher vitamin D levels, promoting a beneficial gut microbiota structure [207,208].

In osteoporosis management, vitamin D enriches beneficial gut bacteria, including butyrate producers [209]. This increases levels of metabolites like butyrate, which acts synergistically with an anti-inflammatory environment to directly promote osteoblast activity and inhibit osteoclast formation. Concurrently, these microbial metabolites (e.g., SCFAs) activate vitamin D receptor (VDR) gene transcription [78]. Together, these effects create a positive feedback loop that enhances bone formation and suppresses resorption, ultimately preventing osteoporosis.

Within the conceptual framework of the gut–bone axis, probiotics, prebiotics, and synbiotics have emerged as key microbiota-directed strategies. Evidence indicates that probiotics not only modulate vitamin D levels [210] but also, as exemplified by *Lactobacillus rhamnosus* GG (LGG), enhance vitamin D absorption to orchestrate bone metabolism [211]. Proposed mechanisms include correcting microbial dysbiosis, thereby improving vitamin D bioavailability [212]. While robust clinical evidence for synbiotics is still accumulating, their capacity to synergistically modulate both vitamin D physiology and the gut microbiome suggests they may outperform single interventions, holding substantial promise for future clinical translation.

Crucially, clinical observations caution against vitamin D over-supplementation, which paradoxically may disrupt microbial equilibrium [213]. However, these findings demand prudent interpretation rather than undue concern. Intriguingly, a randomized controlled trial documented that vitamin D supplementation specifically reduced Bacteroides abundance exclusively in subjects with baseline 25(OH)D ≥ 50 nmol/L (0.89-fold difference; 95% CI 0.81–0.98, *p* = 0.13) [201].

Variations in the vitamin D-microbiota axis related to age and sex are particularly significant. Older adults have more severe vitamin D deficiency and osteoporosis, largely due to age-related gut dysbiosis [214], which may compromise probiotic efficacy and require more intensive intervention. Sex is another key variable; vitamin D’s muscular benefits depend on sufficient testosterone and are modestly affected by estrogen decline [215], effects again linkable to sex hormones and gut microbiota.

Consequently, there is an urgent need for future studies to elucidate how these individual factors, especially age and sex, precisely modulate the vitamin D-gut microbiota interaction network. This knowledge is crucial to underpin the development of personalized gut–bone axis interventions tailored to individual patient profiles.

## 5. Conclusions and Prospects

Bioactive molecules are established key players in gut-mediated osteoporosis. Their levels are dynamically shaped by environmental and physiological factors such as temperature, inflammation, diet, and the gut microenvironment. These compounds offer substantial regulatory potential, making their bioavailability a promising target for intervention. The gut microbiota, a central link between systemic metabolism and bone integrity, interacts closely with these molecules [216]. Microbial metabolites like SCFAs and vitamins affect bone by modulating immune inflammation responses and critical signaling pathways such as RANKL/OPG. Thus, the crosstalk among bioactive molecules, gut microbiota, and bone health is a vital research frontier. Future strategies should target multi-pathway synergy—for example, SCFA-enhanced estrogen signaling or vitamin D-driven microbial modulation—to develop holistic therapies that integrate nutritional and hormonal regulation.

When investigating individual metabolic links to osteoporosis, research should account for sex and age differences. In females, estrogen is a well-established factor, whereas in males, SCFAs and vitamin D metabolism deserve further attention. Older adults also exhibit greater susceptibility to gut microbiota dysbiosis, partly tied to hormonal decline. These aspects warrant careful integration into future studies.

First, SCFA research remains predominantly confined to animal models, while limited clinical data hinder a precise understanding of their biological mechanisms in humans. In particular, the impact of SCFAs on human gut microbiota diversity and composition requires deeper investigation. Large-scale human cohort studies are urgently needed to comprehensively evaluate how demographics, dietary patterns, climate variations, and environmental factors collectively shape gut microbiota-derived metabolites like SCFAs.

Second, estrogen-containing HRT warrants cautious use in individuals with skeletal disorder risk due to elevated breast cancer and cardiovascular complications [143,217]. Furthermore, present investigations into PMO and gut microbiota relationships predominantly employ fecal samples as microbial proxies. This approach may obscure critical variations in microbial communities along different intestinal segments, potentially compromising the accuracy of estrogen’s documented impacts on gut microbiota composition [67]. At the same time, regarding vitamin D, it is crucial to emphasize the variability in gut microbiota abundance observed among populations from different regions, lifestyles, and degrees of vitamin D deficiency following supplementation [201]. This interplay between the biomolecules and intestinal flora represents a dynamic, evolving process rather than a static phenomenon. Studies show gut microbiota remain stable with short-term interventions. Long-term observation is needed for changes to emerge [65]. Long-term interventions trigger adaptive microbial shifts that reciprocally influence bioactive molecules, gradually establishing systemic equilibrium. However, current evidence fails to conclusively establish the temporal dynamics of this bidirectional interaction.

Fecal Microbiota Transplantation (FMT) is widely employed to investigate the gut microbiota’s role, particularly in osteoporosis research. However, microbial loss during FMT can compromise reliability, potentially yielding insignificant outcomes. Stabilizing allogeneic gut microbiota remains a key challenge in current FMT studies. Most mouse recipients rarely sustain homeostasis resembling the transplanted microbial community. Furthermore, FMT research often suffers from limited sample sizes and delayed dynamic monitoring. Optimizing transplantation through advanced measurement and delivery techniques to enhance recipient microbial stability is thus a crucial next step.

The symbiotic relationship between gut bacteria and viruses deserves significant attention. The gut microbiota, a broader ecosystem, encompasses bacteria and bacteriophages that collectively shape the intestinal environment, influencing skeletal health. These components may play synergistic roles in osteoporosis pathogenesis. Gut viruses modulate microbial communities by selectively lysing bacterial hosts, thereby altering the availability of pro- and anti-inflammatory bacterial metabolites critical to bone metabolism. These interactions operate through interdependent mechanisms [14]. Consequently, gut bacteria, viruses, and archaeal communities require comprehensive assessment—an oversight in many studies that neglects their interconnected relationships [218].

Clinical evidence for probiotics alleviating osteoporosis remains limited. Probiotics show no benefit against alveolar bone destruction in non-OVX or non-inflammatory rats [219]. Human trials also report disappointing BMD outcomes; only a few studies demonstrate efficacy in postmenopausal women, raising concerns about probiotic overuse in healthy populations [220]. Inconsistent results for PMO may stem from strain variations, intervention duration, age, or years post-menopause. Stricter trial criteria (e.g., limiting post-menopausal years) and extended interventions are needed [221], alongside optimized probiotic formulations and delivery methods.

Several limitations exist in current research. From a design standpoint, the effect of participant genetics on estrogen metabolism cannot be ruled out, confounding intervention outcomes. Moreover, most research focuses on bone density and turnover markers, while direct evidence linking vitamin D and SCFAs to gut barrier integrity—a more causal endpoint—remains scarce. From an outcomes perspective, clinical data are predominantly derived from postmenopausal women, with male patients underrepresented. Furthermore, for many potential osteoporosis therapies, the tolerable doses and health benefit thresholds are poorly defined, resulting in a lack of personalized recommendations for diverse populations.

Despite these challenges, bioactive metabolites are gaining attention in gut–bone axis research. These microbial compounds show therapeutic potential through two main routes: direct supplementation or indirect modulation via diet or light exposure (e.g., vitamin D synthesis). Both strategies offer promise in targeting osteoporosis through metabolic interventions. This shift is transforming treatment approaches and paving the way for personalized therapies. Emerging technologies are now helping to address existing limitations. For example, nanocoated probiotics and synbiotic microspheres enhance probiotic survival and sustained efficacy, while metagenomic relationship analysis enables precise identification of functional bacterial strains and their metabolites [222,223,224]. These advances provide powerful tools for further exploration of the gut–bone axis.

## Data Availability

Data sharing is not applicable to this article as no new data were created or analyzed in this study.

## Abbreviations

SCFAs	short-chain fatty acids
BMD	bone mineral density
OVX	ovariectomized
ORX	orchiectomized
OP	osteoporosis
HC	healthy controls
PA	propionic acid
MS	multiple sclerosis
RANKL	Receptor Activator of Nuclear Factor Kappa-B Ligand
PXR	Pregnane X Receptor
GPCRs	G protein-coupled receptors
FFAR2/GPR43	free fatty acid receptor 2
LPS	lipopolysaccharide
HIO	high-fat diet-induced obese
NO	non-obese
PTH	parathyroid hormone
IGF-1	insulin-like growth factor-1
GF	germ-free
CaBP	calbindin-D9k
TRPV6	transient receptor potential vanilloid 6
TBP	Tuna Bone Powder
GNPs	Gold Nanoparticles
scFOS	short-chain fructooligosaccharides
NF-κB	nuclear factor kappa-light-chain-enhancer of activated B cells
FOS	fructooligosaccharide
GOS	galactooligosaccharides
HFD	high-fat diet
DIO	disuse-induced osteoporotic
EDCs	endocrine-disrupting chemicals
ZEA	zearalenone
ER	estrogen receptor
HPG	hypothalamic–pituitary–gonadal
HRT	hormone replacement therapy
PMO	postmenopausal osteoporosis
IL-10	interleukin-10
LCA	lithocholic acid
VDR	vitamin D receptor
UV	ultraviolet
FMT	fecal microbiota transplantation
